# Integrated lncRNA and mRNA Transcriptome Analyses of IGF1 and IGF2 Stimulated Ovaries Reveal Genes and Pathways Potentially Associated with Ovarian Development and Oocyte Maturation in Golden Pompano (*Trachinotus ovatus*)

**DOI:** 10.3390/ani15081134

**Published:** 2025-04-15

**Authors:** Charles Brighton Ndandala, Yuwen Guo, Zhimin Ju, Muhammad Fachri, Happiness Moses Mwemi, Huapu Chen

**Affiliations:** 1 Guangdong Research Center on Reproductive Control and Breeding Technology of Indigenous Valuable Fish Species, Guangdong Provincial Key Laboratory of Aquatic Animal Disease Control and Healthy Culture, Fisheries College, Guangdong Ocean University, Zhanjiang 524088, China; charlesndandala@gmail.com (C.B.N.); gdougyw@163.com (Y.G.); j1274808741@gmail.com (Z.J.); muhammadfachri185@gmail.com (M.F.); happinesstomi13@gmail.com (H.M.M.); 2 Southern Marine Science and Engineering Guangdong Laboratory (Zhanjiang), Zhanjiang 524025, China

**Keywords:** IGFs, transcriptome, lncRNA, ovary, reproduction, *Trachinotus ovatus*

## Abstract

Insulin-like growth factors (IGFs) play crucial roles in the regulation of animal growth and reproduction. However, the functional and regulatory mechanisms underlying ovarian growth and oocyte maturation in teleosts remain unclear. In this study, the expression profiles of lncRNAs and mRNAs were analyzed in the ovaries of golden pompano (*Trachinotus ovatus*) treated with IGF1 and IGF2 proteins to gain insights into the role of these two IGF ligands in the regulation of ovarian development and maturation. A total of 1494 lncRNAs and 8728 mRNAs were differentially expressed following IGF1 treatment compared with the control group. A total of 101 lncRNAs and 377 mRNAs were differentially expressed after IGF2 treatment compared to those in the control group. The results revealed that KEGG pathways enriched by target genes of the DE lncRNAs overlapped significantly with those enriched by the DE mRNAs in both the IGF1 and IGF2 groups. The key overlapping enriched pathways included ECM receptor interaction, gap junction, Hedgehog signaling pathway, Ras signaling pathway, Rap1 signaling pathway, TGF beta signaling pathway, Wnt signaling pathway, GnRH signaling pathway, progesterone-mediated oocyte maturation, oocyte meiosis, cell cycle, and MAPK signaling pathway. The differentially expressed genes (DEGs) involved in ovarian development and oocyte maturation were *cyp17a1*, *cyp19a1*, *star*, *hsd17b3*, *hsd17b7*, *adam23*, *slc26a6*, *htr2b*, *h2ax*, *nanos3*, *krt18*, *pgr*, and *inhbb*, following IGF1 and IGF2 treatment. Furthermore, four lncRNAs (MSTRG.66521.1, MSTRG.49969.1, MSTRG.59923.1, and MSTRG.13767.1) for IGF1 and two (MSTRG.20896.2 and MSTRG.58123.2) for IGF2 within the lncRNA–mRNA network were found to target DEGs related to ovarian development and maturation. This suggests that IGFs may affect reproductive processes by regulating the expression of lncRNAs and mRNAs. RT-qPCR analysis revealed that these six lncRNAs showed high expression levels in the brain, pituitary, liver, and gonad tissues, indicating their potential involvement in regulating ovarian growth and development. This study elucidates the lncRNA–mRNA regulatory mechanism in response to IGF1 and IGF2 treatment during stage III of ovarian development in golden pompano, thereby deepening our understanding of its functional role.

## Simple Summary

The golden pompano (*Trachinotus ovatus*) is an economically important marine fish species in aquaculture because of its rapid growth, mild flavor, and nutrient-rich flesh. Ovarian development is a critical determinant of high-quality egg production and aquaculture productivity. However, the role of the IGF axis in regulating ovarian maturation in this species remains poorly understood. This study investigated molecular responses to IGF1 and IGF2 stimulation by identifying the genes and pathways involved in ovarian maturation. The findings aim to uncover the molecular mechanisms driving reproduction in golden pompano. This study provides foundational insights into optimizing aquaculture productivity through targeted breeding strategies.

## 1. Introduction

Ovarian development is a complex biological process involving numerous critical signaling pathways and gene regulatory mechanisms [1,2,3]. The process requires precise coordination of various molecular factors, including hormones, growth factors, and transcription factors, to ensure proper folliculogenesis, oocyte maturation, and ovulation [4]. Additionally, factors such as the IGF system, which regulates cellular growth, differentiation, and survival, play key roles in ovarian function and reproductive health [5]. Understanding the molecular and cellular dynamics involved in ovarian development is essential for advancing reproductive biotechnology and improving the management of animal breeding, particularly in aquaculture species [6,7,8].

Complex intraovarian IGF systems have been identified in fish, including spotted scats [2], rainbow trout [9], medaka [10], and zebrafish [11]. These systems comprise multiple ligands, such as IGF1, IGF2a, IGF2b, and IGF3, as well as receptors (IGF1R, IGF1R1, and IGF1R2) and IGF-binding proteins (IGFBPs) [8]. Although teleost fish serve as important models for studying ovarian development and the IGF system, there is limited information on the role of IGFs in ovarian development in golden pompano. The intraovarian expression profiles of the IGF system suggest its involvement in regulating ovarian development in teleosts [12]. In rainbow trout, as follicles mature, the expression of IGF1 and IGF2 transcripts increases in the gonads, similar to a previous study, while igfbp3 decreases, and igfbp6 and IGFBP-related protein-1 (igfbp-rP1) increase [13]. In coho salmon (*Oncorhynchus kisutch*), IGF1 binding was observed in both theca-interstitial and granulosa cell layers of the ovarian follicle [13,14].

Several studies have highlighted the specific physiological functions of IGFs in fish ovary [8,13,15,16,17,18]. IGF1 and IGF2 have been shown to enhance thymidine incorporation in the vitellogenic follicles of goldfish while also facilitating oocyte maturation in species such as rainbow trout, red seabream, mummichog, and shortfinned eel [17,19]. In vitro analysis of rainbow trout ovaries revealed that IGF1 mRNA levels did not significantly change during the acquisition of follicular maturational competence (FMC); IGF2 mRNA levels were significantly elevated in females exhibiting high FMC [12,20]. This suggests a functional role of IGF2 in folliculogenesis. This aligns with findings in mummichog, where IGF2 stimulates germinal vesicle migration and breakdown (GVBD), which is an indicator of meiosis resumption [21]. Notably, IGF1 has a more immediate and effective impact on oocyte maturation than 17α, 20β-dihydroxy-4-pregnen-3-one, which is the principal maturation-inducing steroid hormone (MIH) produced by the interaction of the theca and granulosa cell layers [22].

Long noncoding RNAs (lncRNAs) are transcripts with a total length of more than 200 nucleotides that do not encode proteins [23]. They are predominantly found in the nucleus, where they often outnumber the mRNAs. LncRNAs perform various biological functions by acting on protein-coding genes in cis and trans forms [24]. They are involved in numerous biological processes, such as germ cell growth, meiosis, gamete production, and sex differentiation [25,26]. Many studies have highlighted the crucial role of lncRNAs in reproductive development in various organisms [25,26,27,28,29]. LncRNAs interact with mRNAs, DNA, or proteins to regulate transcription, post-transcriptional modification, and translation, influencing cellular activities and physiological processes [30,31,32,33]. Although studies have demonstrated that lncRNAs play key roles in growth, energy metabolism, and reproduction, there is still little information on the molecular mechanisms by which lncRNAs regulate ovarian growth, development, and maturation in the golden pompano.

*Trachinotus ovatus*, commonly known as golden pompano, belongs to the Perciformes order and family Carangidae [34]. It is mainly cultivated along the southern coast of China because of its economic value [35,36]. However, achieving sexual maturity in captivity takes approximately three to four years, which greatly increases both the cost and risks of aquaculture. Our previous study highlighted the role of the IGF system in promoting growth and the reproductive system [34], but its specific involvement in reproductive processes remains poorly understood. Therefore, advancing research on the regulation of reproduction in golden pompano in response to the IGF system is essential for gaining a deeper understanding of the species’ endocrine regulatory network. This knowledge could potentially optimize breeding strategies and reduce the time and costs associated with aquaculture.

In the present study, RNA sequencing was performed to identify specific lncRNAs and mRNAs expressed in *T. ovatus* ovaries at stage III of development in response to IGF1 and IGF2 recombinant protein stimulation in vitro. Differentially expressed genes were identified, a protein–protein interaction (PPI) network was constructed, and key regulatory mechanisms involving lncRNAs and mRNAs related to ovarian development and maturation were identified. Given the economic and nutritional value of *T. ovatus* in aquaculture, challenges remain in its genetic breeding. Therefore, this study enhances our understanding of the physiological functions of IGFs and identifies prospective intervention targets for genetic enhancement in aquaculture.

## 2. Materials and Methods

### 2.1. Ethics Statement

All experiments were approved by the Animal Research and Ethics Committees of the Fisheries College of Guangdong Ocean University, Zhanjiang, Guangdong, China (ethical batch number: GDOU-IACUC-2021-A1229). All efforts were made to minimize pain.

### 2.2. Sample Treatment

First, we obtained recombinant IGF1 and IGF2 proteins; briefly, recombinant IGF1 and IGF2 proteins were produced by amplifying the *T. ovatus* coding sequence [34] using primers designed with Primer Premier 6 (Table S1). His-SUMO-tagged IGF1 (Wuhan GeneCreate Biological Engineering Co., Ltd., Wuhan, China) was cloned into the pET-SUMO vector; glutathione S-transferase (GST)-tagged IGF2 (LMAIBio, Shanghai, China) was cloned into the pGEXT-4T1 vector. Constructs were transformed into *Escherichia coli* rosetta2 (DE3) (Huamei Biotechnology, Wuhan, China) and cultured in 200 mL Luria–Bertani (LB) medium supplemented with antibiotics (IGF1: 50 µg/mL ampicillin and 50 µg/mL kanamycin; IGF2: 100 µg/mL ampicillin and 50 µg/mL chloramphenicol) at 37 °C until the OD_600_ reached 0.6–0.8. Protein was induced with 1 mM isopropyl β-D-thiogalactoside (IPTG, YEASEN, Shanghai, China) for 4–5 h at 37 °C, followed by cell lysis via sonication in Tris-HCl buffer (pH 8.0, 0.5 M NaCl). Proteins were purified using Ni-NTA affinity chromatography [37], and purity was confirmed by 12% SDS-PAGE.

**Figure 1:**
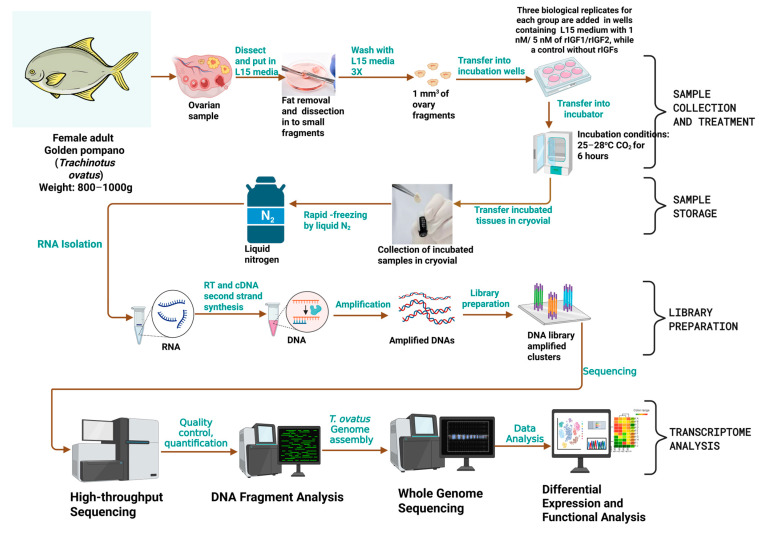
Schematic representation of the in vitro experimental design, including sample treatment and the RNA sequencing workflow.

Ovary samples were collected from six female *T. ovatus* with a body weight of 900 ± 20 g (mean ± SEM, n = 6). The in vitro incubation of ovarian tissue was performed according to the method described in our previous study [23], as shown in Figure 1. The treatment group was supplemented with a medium containing 1 nM and 5 nM of IGF1 and IGF2 recombinant proteins, respectively, while the control group was incubated without IGF1 and IGF2 recombinant proteins (n = 6 for each group). After 6 h of incubation, the samples were collected and stored at −80 °C for RNA extraction. Additionally, tissue samples were collected from three female (n = 3) and three male (n = 3) golden pompano. Twelve distinct tissues were isolated from each fish: brain, pituitary, gill, heart, liver, kidney, spleen, stomach, intestine, muscle, ovary (from females), and testis (from males). Each tissue type was collected in triplicate for subsequent RNA isolation.

### 2.3. RNA Library Construction and Sequencing

RNA extraction was performed using TRIzol reagent (Invitrogen, Carlsbad, CA, USA), according to the manufacturer’s protocol. Briefly, tissues were homogenized in TRIzol, followed by phase separation with chloroform, RNA precipitation with isopropanol, and washing with ethanol. The purified RNA was dissolved in RNAse-free water. RNA concentration was quantified using a Qubit 3.0 (Thermo Fisher Scientific, Waltham, MA, USA) with RNA-specific fluorescent dyes to ensure accuracy, and RNA integrity was assessed using an Agilent 2100 (Agilent Technologies, Santa Clara, CA, USA), which generates an RNA Integrity Number (RIN; 1–10 scale) via microfluidic capillary electrophoresis. Samples with RIN > 7.0 were retained for downstream analysis, and RNA purity was confirmed by 1% agarose gel electrophoresis to visualize intact 28S/18S rRNA bands and rule out degradation or contamination. After quality control, the RNA was used to construct an RNA library. Initially, rRNA was removed using the Ribo-Zero Gold Kit (Epicentre Technologies, Madison, WI, USA), and the RNA was fragmented randomly using a fragmentation buffer. The first strand of cDNA was synthesized from the fragmented RNA, followed by synthesis of the complementary cDNA strand. After purification with VAHTS DNA Clean Beads (Vazyme, Nanjing, China), double-stranded cDNA was used for end repair, A-tailing, and sequencing adaptor ligation, resulting in the generation of cDNA library by PCR amplification. After confirmation of purity and quality, sequencing was performed using Illumina NovaSeq X Plus by Gene Denovo Biotechnology Co. (Guangzhou, China).

### 2.4. Sequencing Quality Control and Analysis

Raw sequencing data containing reads with adapter sequences, low-quality bases (*q*-value ≤ 20), or more than 50% unknown nucleotides (N) were filtered to produce clean data. These clean datasets were then aligned to the golden pompano genome (10.6084/m9.figshare.7570727.v3) using HISAT2 software version 2.2.0 [38] to obtain the mapped data. The mapped reads of each sample were assembled using StringTie (v1.3.0) [39]. Quality analysis and subsequent evaluation of the sequencing library were performed based on the mapped data, which included tests for insert length and randomness (Figure 1).

### 2.5. lncRNA Identification and Prediction

Candidate lncRNAs were identified using CNCI (version 2) https://github.com/www-bioinfo-org/CNCI, accessed on 22 February 2025, CPC (version 0.9-r2) (http://cpc.cbi.pku.edu.cn/), and FEELNC (version v0.2) (https://github.com/tderrien/FEELnc, accessed on 22 February 2025), with the requirements that they are longer than 200 nucleotides and contain more than two exons. The lncRNAs were predicted for cis- and trans-target genes. Cis-target genes were identified based on their location within 100 kb regions upstream or downstream of the lncRNAs. Trans-target genes were identified by comparing the abundance of lncRNAs with known protein-coding genes in the treated and control groups using Pearson’s correlation coefficient method (|r| > 0.8, *p* < 0.01).

### 2.6. Identification of DE lncRNAs and mRNAs

Differentially expressed genes coding for lncRNAs and mRNA were analyzed using the DEseq2 package version 1.46.0 [40] to identify significant differences between the two groups. Genes with a parameter fold change (FC) ≥ 1.5 and *p*-value < 0.05 were considered differentially expressed genes.

### 2.7. Functional Enrichment Analysis

Functional enrichment analysis of target genes of the DE lncRNAs and DE mRNAs, including Gene Ontology (GO) and Kyoto Encyclopedia of Genes and Genomes (KEGG) pathway analyses, were performed by comparing the gene ontology database (http://www.geneontology.org/ accessed on 23 February 2025) and whole genome background. To assess statistical significance, we used a *p*-value < 0.05.

### 2.8. PPI Network Construction and Hub Genes Analysis

Differentially expressed genes (DEGs) were used to construct the PPI network using the STRING software version 12.0 [41]. Key clusters within the PPI network were identified using Molecular Complex Detection (MCODE) with a cut-off score threshold of >2 [42]. Hub genes within the network were selected using CytoHubba version 0.1 [43], based on a connection degree threshold of >2. The PPI network was visualized using the Cytoscape software version 3.0 [44].

### 2.9. lncRNA–mRNA Network Construction

To construct a preliminary network of lncRNAs and mRNAs, we focused on DE lncRNAs and mRNAs involved in reproduction and growth. Using the candidate lncRNAs and their target genes, a lncRNA–mRNA co-expression network was constructed and visualized using the Cytoscape software.

### 2.10. Validation by Real-Time Quantitative PCR

Total RNA was reverse-transcribed into cDNA using the PrimeScript™ RT Reagent Kit with gDNA Eraser (Perfect Real Time) (TaKaRa, Kusatsu, Japan), followed by RT-qPCR using PerfectStart^®^ Green qPCR SuperMix (TransGen Biotech, Beijing, China). PCR conditions were as follows: initial denaturation at 95 °C for 2 min, followed by 35 cycles of denaturation at 95 °C for 20 s, annealing at 60 °C for 20 s, and extension at 72 °C for 20 s. Gene expression levels were calculated using the 2^−∆∆Ct^ method with *β-actin* as the housekeeping gene. All primer sequences were designed using Primer Premier 6.0 software, and are listed in Table 1.

### 2.11. Tissue Distribution of Candidate lncRNAs by RT-qPCR

The expression levels of the four candidate lncRNAs (MSTRG.26614.1, MSTRG.98390.1, MSTRG.77400.2, and MSTRG.84457.1) after IGF1 treatment and two candidate lncRNAs (MSTRG.20896.1, and MSTRG.58123.2) after IGF2 treatment were evaluated in different tissues (brain, pituitary gland, heart, gill, kidney, liver, stomach, intestine, spleen, muscle, ovary, and testis) using RT-qPCR. Details of RNA isolation, reverse transcription, and RT-qPCR protocols are described in Section 2.10. The primer sequences used for the lncRNAs are listed in Table 1.

### 2.12. Data Analysis

Statistical analysis was performed using SPSS 20.0 (SPSS, Chicago, IL, USA). One-way ANOVA followed by Tukey’s post-hoc test were used to analyze the significant difference, using a confidence level of *p* < 0.05. Data are expressed as the means ± SEM (n = 3).

## 3. Results

### 3.1. Overview of the RNA-Seq Results

**Figure 2:**
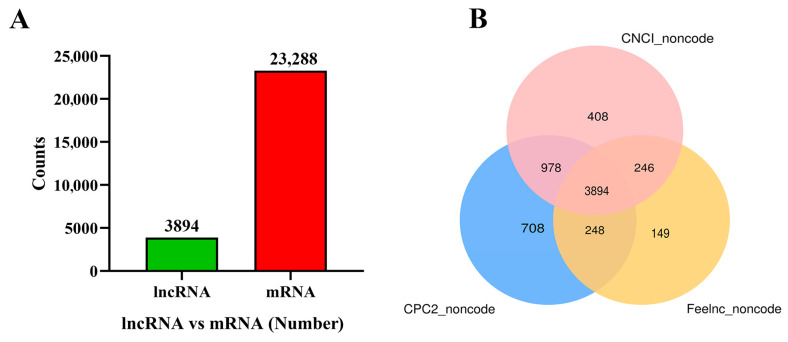
Overview of RNA sequencing. (**A**) Number of lncRNAs and mRNAs. (**B**) Venn diagram according to annotation by CNCI, CPC2, and Feelnc.

Nine cDNA libraries were constructed and sequenced, resulting in 117,589,081,862 clean reads with an average Q30 above 94.91% after quality control. The clean reads were aligned to the golden pompano reference genome with alignment percentages ranging from 90.13% to 93.27% (Table 2). The mapped reads were used for transcript construction, and 3894 lncRNAs and 23,288 mRNAs were identified by CPC, CNCI, and Feelnc in response to IGF1 and IGF2 stimulation, as shown in Figure 2A,B.

### 3.2. Identification of the DE lncRNAs and mRNAs

**Figure 3:**
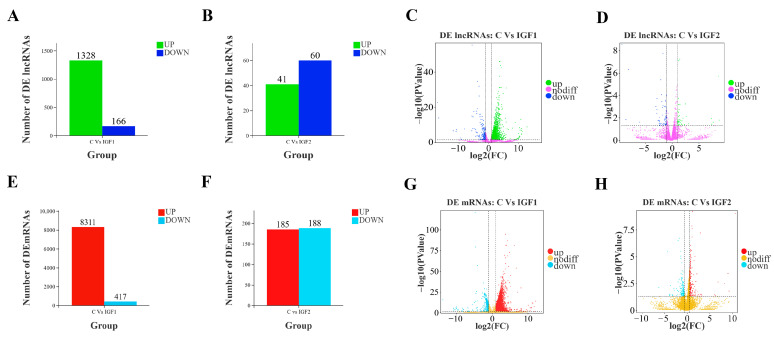
Bar charts and volcano plot figures of the DE lncRNAs and DE mRNAs of the different comparison groups. (**A**): The number of upregulated and downregulated DE lncRNAs between the control and IGF1-treated groups. (**B**): The number of upregulated and downregulated DE lncRNAs between the control and IGF2-treated groups. (**C**): Volcano plot of the DE lncRNAs distribution between the control and IGF1-treated groups. (**D**): Volcano plot of the DE lncRNAs distribution between the control and IGF2-treated groups. (**E**): The number of upregulated and downregulated DE mRNAs between the control and IGF1-treated groups. (**F**): The number of DE mRNAs between the control and IGF2-treated groups. (**G**): Volcano plot of the DE mRNAs levels between the control and IGF1-treated groups. (**H**): Volcano plot of the DE mRNAs levels between the control and IGF2-treated groups. Green, blue, and pink colors represent lncRNAs, red, light blue, and golden colors represent mRNAs.

A total of 1494 lncRNAs and 8728 mRNAs were differentially expressed following IGF1 stimulation compared to the control group, including 1328 upregulated and 166 downregulated lncRNAs (Figure 3A) and 8311 upregulated and 417 downregulated mRNAs (*p* < 0.05, FC ≥ 1.5) (Figure 3B). Following IGF2 treatment, 101 lncRNAs and 377 mRNAs were differentially expressed relative to the control group, comprising 41 upregulated and 60 downregulated lncRNAs (Figure 3F) and 185 upregulated mRNAs and 188 downregulated mRNAs (Figure 3D) (*p* < 0.05, FC ≥ 1.5). Volcano plots illustrate the distribution of upregulated and downregulated lncRNAs and mRNAs: IGF1-treated samples are shown in Figure 3C and Figure 3G for lncRNAs and mRNAs, respectively. IGF2-treated samples are displayed in Figure 3E,H for lncRNAs and mRNAs, respectively.

### 3.3. GO and KEGG Pathway Enrichment Analysis

**Figure 4:**
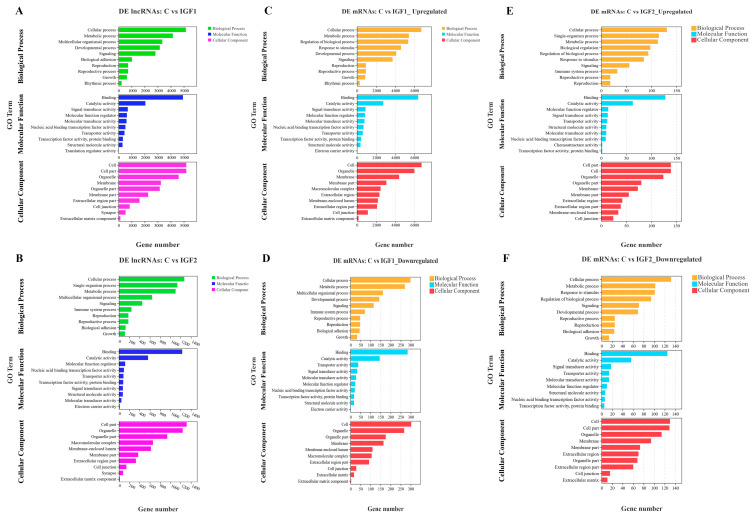
The top 10 GO enrichment analysis terms of the target genes of the DE lncRNAs and DE mRNAs between control and treatment groups. (**A**): DE lncRNAs between the control and IGF1 groups. (**B**): DE lncRNAs between the control and IGF2 groups. (**C**): Upregulated GO terms of the DE mRNAs between the control and IGF1 groups. (**D**): Downregulated GO terms of the DE mRNAs between the control and IGF1 groups. (**E**): Upregulated GO terms of the DE mRNAs between the control and IGF2 groups. (**F**): Downregulated GO terms of the DE mRNAs between the control and IGF2 groups. The terms related to biological process, cellular component, and molecular function for lncRNAs are presented by green, blue, and pink, respectively, while for the mRNAs, they are presented by yellow, light blue, and red, respectively.

To investigate the potential interactions between the DE lncRNAs and DE mRNAs, the target genes of all of the DE lncRNAs were predicted (Table S2). Subsequently, GO enrichment analysis was performed to identify the most significantly enriched GO terms associated with the DE lncRNAs and DE mRNAs in response to the control and IGF1 and IGF2 comparison groups. The results highlighted several enriched terms for biological processes, cellular components, and molecular functions. Similarly, GO enrichment analysis was performed specifically for the DE mRNAs, and the enrichment terms are presented in Figure 4A,B for the IGF1 group and Figure 4C,D for the IGF2 group.

There was a high degree of overlap between the enriched GO terms for the DE lncRNAs and DE mRNAs. Key shared terms in biological processes include cellular process, metabolic process, reproductive process, and biological adhesion. In terms of molecular functions, shared terms include binding, catalytic activity, molecular function regulator, and transporter activities. For cellular components, key overlapping terms included cell junction, extracellular matrix component, and membrane (more details are provided in Table S3). These results indicate that both lncRNA and mRNAs participate in closely related functional pathways, suggesting their potential regulatory roles in reproduction, growth, and hormonal signaling in *T. ovatus* under IGF treatments

**Figure 5:**
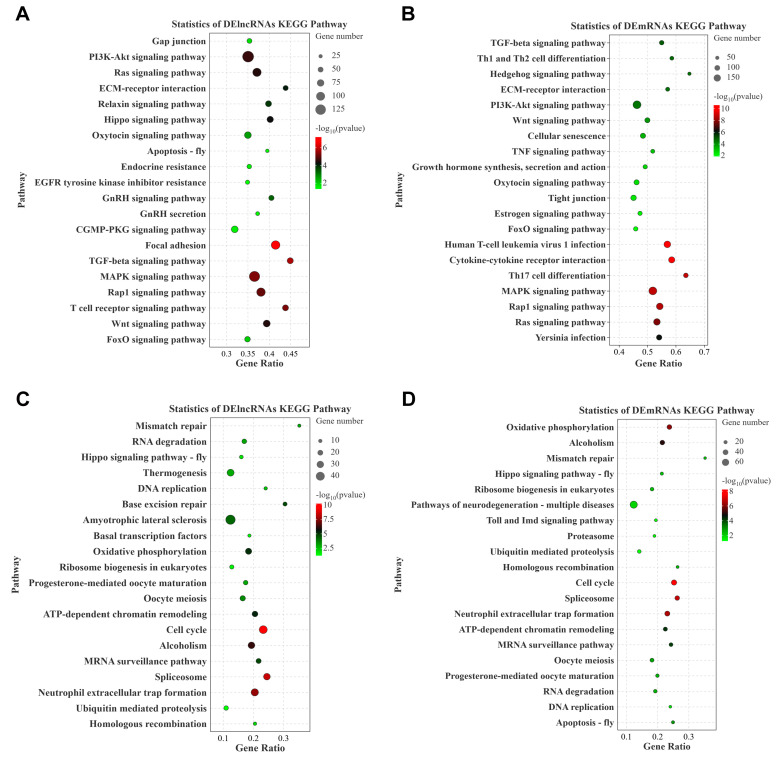
KEGG pathway enrichment analysis of the target genes of the DE lncRNAs and DE mRNAs between different groups. (**A**) DE lncRNAs between control and IGF1, (**B**) DE lncRNAs between control and IGF2, (**C**) DE mRNAs between control and IGF1, (**D**) DE mRNAs between control and IGF2. The spot color represents the q-value of the enriched pathway. Spot size represents the number of genes enriched in each pathway.

KEGG pathway enrichment analysis was conducted to identify the functional pathways associated with the target genes of the DE lncRNAs and DE mRNAs (Table S4). The results revealed that the KEGG pathways enriched by target genes of the DE lncRNAs following IGF1 stimulation (Figure 5A,B) overlapped significantly with those enriched by the DE mRNAs, similar to IGF2 stimulation (Figure 5C,D). The key overlapping pathways included ECM receptor interaction, gap junction, Hedgehog signaling pathway, Ras signaling pathway, Rap1 signaling pathway, TGF beta signaling pathway, Wnt signaling pathway, GnRH signaling pathway, progesterone-mediated oocyte maturation, oocyte meiosis, cell cycle, and MAPK signaling pathway. These enriched pathways and overlapping characteristics highlight the crucial roles of both lncRNAs and mRNAs in regulating ovarian development and maturation, thereby providing valuable insights into the complex molecular mechanisms underlying IGF treatment in *T. ovatus.*

### 3.4. Construction of the PPI Network and the Identification of Hub Genes

#### 3.4.1. The PPI Network Following IGF1 Stimulation

**Figure 6:**
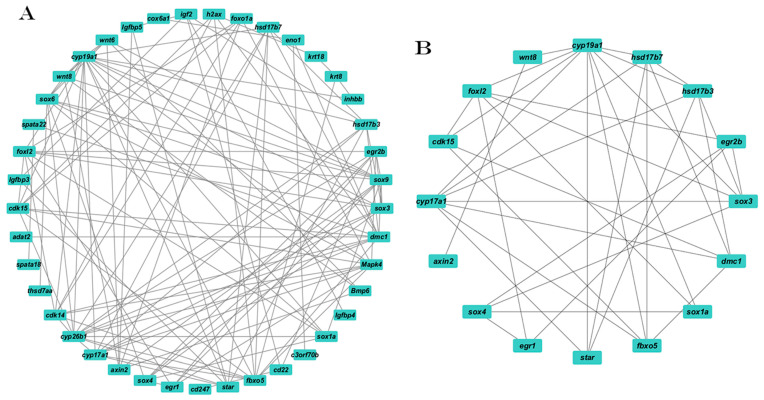
The PPI network of the DEGs between the control and IGF1-treated groups. (**A**) The whole PPI network. (**B**) A specific PPI network focused on the genes related to reproduction.

Based on the STRING analysis results, the PPI network for the control versus IGF1 comparison group comprised 41 nodes and 119 edges (Figure 6A). In addition, a specific PPI network of key genes related to ovarian growth, development, and maturation was constructed, comprising 16 hub genes and 38 edges. This specialized network includes key genes, such as *cyp19a1*, *hsd17b7*, *hsd17b3*, *egr2b*, *soc3*, *dcm1*, *sox1a*, *fbxo5*, *star*, *egr1*, *sox4*, *axin2*, *cyp17a1*, *cdk15*, *foxl2*, and *wnt8* (Figure 6B).

#### 3.4.2. The PPI Network Following IGF2 Stimulation

**Figure 7:**
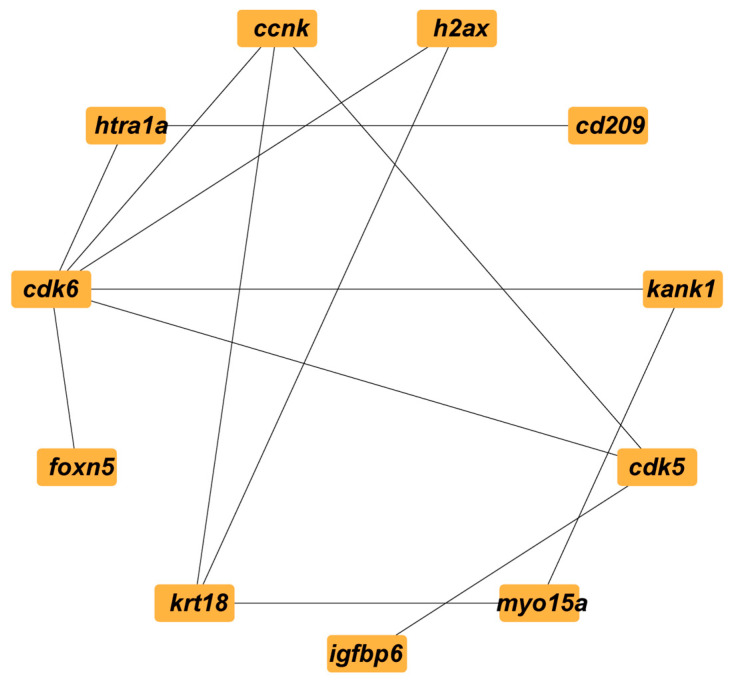
The PPI network of the target DEGs related to ovarian development and oocyte maturation between the control and IGF2-treated groups.

Based on the STRING analysis results for the control versus IGF2 comparison group candidate genes, a PPI network was constructed comprising 11 nodes and 13 edges, such as *ccnk*, *h2ax*, *kank1*, *cdk5*, *cd209*, *myo15a*, *igfbp6*, *krt18*, *cdk6*, *foxn5*, and *htra1a* (Figure 7). The analysis identified 11 hub genes associated with direct and indirect ovarian growth and development.

### 3.5. Construction of the lncRNA–mRNA Regulatory Network

#### 3.5.1. The IGF1 lncRNA–mRNA Regulatory Network

**Figure 8:**
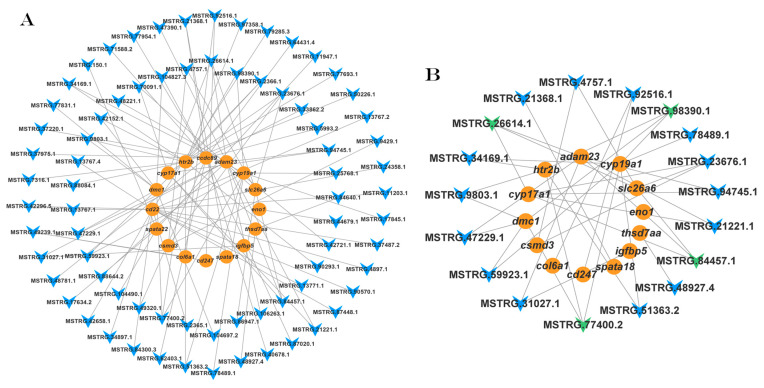
Co-expression network of the DE lncRNAs and mRNAs. (**A**) Network map comprising 74 lncRNAs and 16 mRNAs. (**B**) Sub-network of the lncRNAs (MSTRG.66521.1, MSTRG.49969.1, MSTRG.59923.1, and MSTRG.13767.1). Blue arrow-shaped nodes represent lncRNAs, green arrow-shaped nodes represent target lncRNAs, and orange circular-shaped nodes represent mRNAs.

To investigate the potential regulatory roles of lncRNAs and mRNAs in the ovary following IGF1 treatment, DE mRNAs associated with ovarian growth and maturation, along with the associated DE lncRNAs were randomly selected to construct a lncRNA–mRNA co-expression regulatory network. This network comprised 74 lncRNAs and 16 mRNAs (Figure 8A). Within this network, four lncRNAs (MSTRG.66521.1, MSTRG.49969.1, MSTRG.59923.1, and MSTRG.13767.1) were identified as target DE mRNAs related to ovarian growth and maturation (Figure 8B). These findings suggest that these lncRNAs may play significant roles in regulating ovarian development and maturation by modulating the expression of important genes in response to IGF1 treatment.

#### 3.5.2. The IGF2 lncRNA-mRNA Regulatory Network

**Figure 9:**
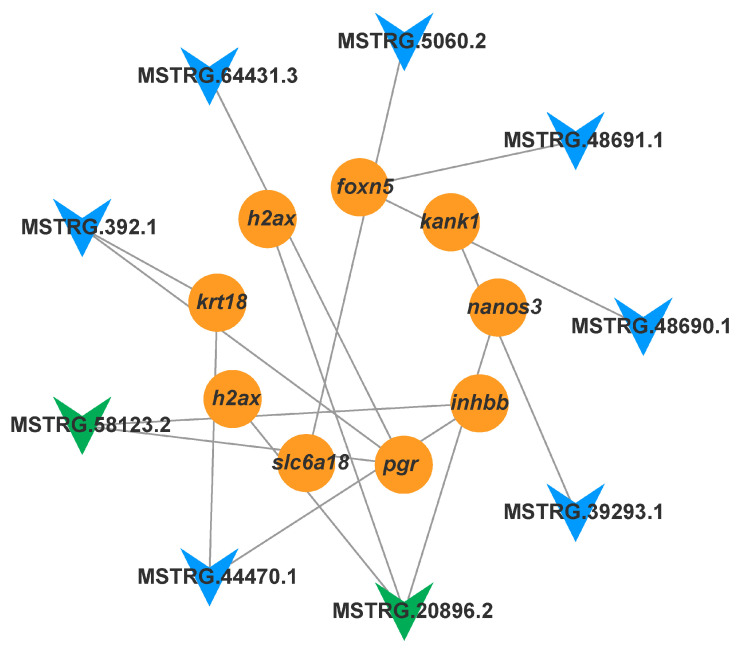
Co-expression network of the DE lncRNAs and mRNAs in response to IGF2 stimulation. The network map includes nine lncRNAs and nine mRNAs. Blue arrow-shaped nodes represent lncRNAs, green arrow-shaped nodes represent target lncRNAs (MSTRG.20896.2 and MSTRG.58123.2), and orange circular-shaped nodes represent mRNAs.

To explore the potential regulatory roles of lncRNAs and mRNAs in the ovary under IGF2 treatment, DE mRNAs associated with ovarian growth and maturation, along with the targeted DE lncRNAs, were selected to construct a lncRNA–mRNA co-expression network (Figure 9). This network included nine lncRNAs and nine mRNAs. Among these, two lncRNAs (MSTRG.20896.2 and MSTRG.58123.2) were identified as key regulators, targeting key DEGs related to ovarian growth and reproductive processes. These findings suggest that these specific lncRNAs may play critical roles in regulating the expression of reproductive and growth-related genes in the ovary, potentially influencing ovarian development and maturation.

### 3.6. Validation by RT-qPCR

**Figure 10:**
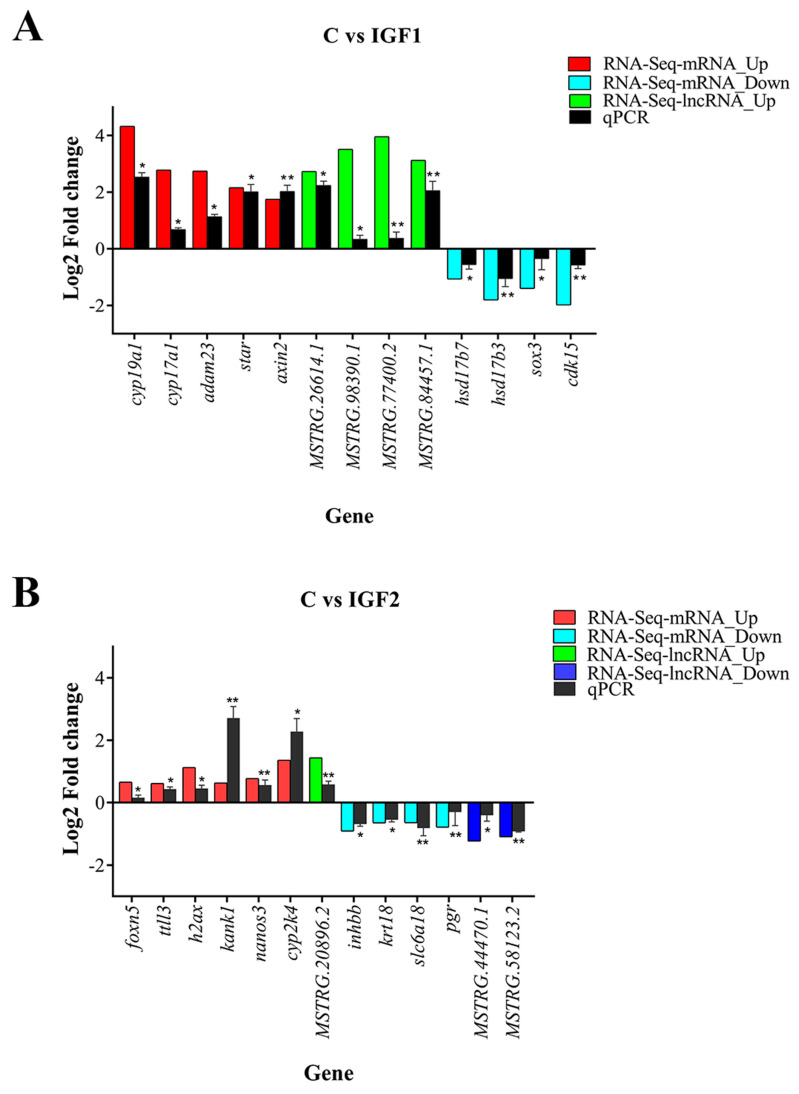
Validation of RNA-Seq results by RT-qPCR. Relative expression levels were measured using the 2^−∆∆Ct^ method, normalized to the reference gene *β*-actin. Data are presented as mean ± standard error (SEM) (n = 3). One-way analysis of variance (ANOVA) followed by Tukey’s post hoc test was used to test the significance difference between control and treated groups at *p* < 0.05. * indicates *p* < 0.05 and ** indicates *p* < 0.01. (**A**) Validation of the nine DE mRNAs and four DE lncRNAs in control versus IGF1-treated groups. (**B**) Validation of 10 DE mRNAs and three DE lncRNAs in the control versus IGF2-treated group. Green and blue colors represent lncRNAs, while red and light blue colors represent mRNAs. The black color represents qPCR.

To validate the RNA-Seq results, DE lncRNAs and DE mRNAs in response to IGF1 and IGF2 treatment were randomly selected for RT-qPCR analysis. The validation results showed that the expression trends for all nine DE mRNAs and four DE lncRNAs in the IGF1 comparison group were consistent with the RNA-Seq results (Figure 10A). Similarly, in the IGF2 comparison group, the expression trends for all 10 DE mRNAs and three DE lncRNAs matched the RNA-Seq results (Figure 10B). These consistent results from both RNA-Seq and RT-qPCR analyses confirmed the accuracy and readability of the RNA-seq data, providing further support for the identified gene expression changes in response to the IGF treatments.

### 3.7. Tissue Distribution of the Candidate lncRNAs

**Figure 11:**
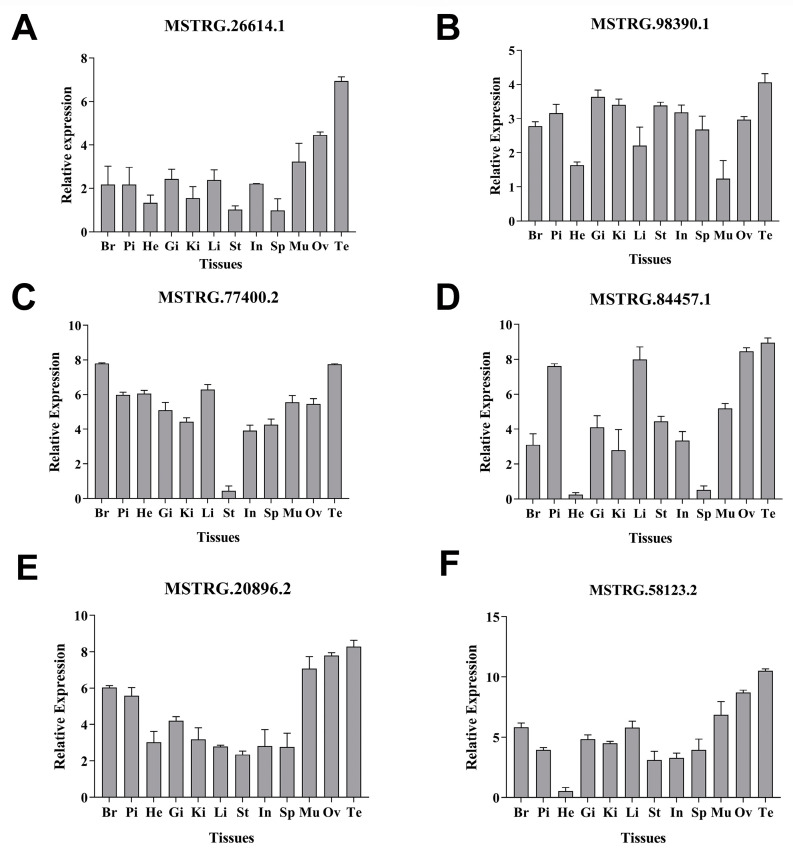
Tissue distribution of the lncRNAs. (**A**) MSTRG.26614.1, (**B**) MSTRG.98390.1, (**C**) MSTRG.77400.2, (**D**) MSTRG.84457.1, (**E**) MSTRG.20896.2, and (**F**) MSTRG.58123.2. Br, brain; Pi, pituitary; He, heart; Gi, gill; Ki, kidney; Li, liver: St, stomach; In, intestine; Sp, spleen; Mu, muscle; Ov, ovary; and Te, testis. Data are expressed as mean ± SEM (n = 3).

The expression distribution of the four candidate lncRNAs (MSTRG.26614.1, MSTRG.98390.1, MSTRG.77400.2, and MSTRG.84457.1), which target DEGs related to ovarian growth and development, was analyzed across 12 tissue types of golden pompano in the control versus IGF1 comparison groups using RT-qPCR. The results revealed that these four lncRNAs exhibited high expression levels in the brain, pituitary gland, liver, and gonads (Figure 11A–D). Furthermore, the expression distribution of the two lncRNAs (MSTRG.20896.2 and MSTRG.58123.2), which also target DEGs related to ovarian growth and development, was investigated across the same 12 tissue types in the control versus IGF2 comparison group. The results indicate that these lncRNAs were highly expressed in the brain, pituitary gland, and gonads (Figure 11E,F). These findings suggest the potential involvement of these lncRNAs in regulation of ovarian development and maturation in golden pompano through the action of IGFs.

## 4. Discussion

Ovarian development and maturation are governed by complex interactions between gene expression and environmental factors underpinned by intricate molecular regulatory networks [13,45,46]. IGFs play a vital role in various physiological processes, including embryonic development, sex differentiation, metabolism, immune responses, circadian rhythms, and stress adaptation in teleosts [47,48,49]. The IGF system reveals the complex mechanisms involved in ovarian growth, development, and maturation, which are initiated by the production of Müllerian Inhibiting Hormone (MIH) by the post-vitellogenic follicle complex [50,51]. Research has shown that IGFs can induce GVBD in vitro during the earlier stages of follicle development in many fish species [13]. In striped bass [48] and zebrafish [52], IGF1 has been shown to induce GVBD, even in maturation-inducing steroid (MIS) incompetent follicles [13,48]. However, in rainbow trout, in vitro treatment with either IGF1 or IGF2 did not induce GVBD, which is typically used as a marker for oocyte resumption [13]. Furthermore, when IGF1 peptide was co-incubated with MIS, it did not induce GVBD, suggesting that IGFs alone cannot induce oocyte maturational competence (OMC) as the ability of oocytes to respond to MIS [13]. Findings from previous studies suggest that the involvement of IGFs in regulating OMC has received less attention than their role in inducing oocyte meiosis resumption [20,22,53].

Understanding the complex regulatory mechanisms governing reproduction and growth in teleosts, particularly those governing ovarian growth, development, maturation, and ovulation, is crucial for the development of the aquaculture industry [54]. RNA-Seq analysis was performed to assess the lncRNA regulatory mechanisms involved in reproductive processes and identify key genes and transcriptomic changes at stage III of *T. ovatus* ovarian development in response to IGF1 and IGF2 protein treatment in vitro. In this study, we identified 1328 upregulated and 166 downregulated DE lncRNAs, while 8311 upregulated and 417 downregulated DE mRNAs were identified in the IGF1 group compared to the control group. In response to IGF2 stimulation, we identified 41 upregulated and 60 downregulated DE lncRNAs and 185 upregulated and 188 downregulated DE mRNAs. These findings provide insights into the transcriptomic landscape and molecular mechanisms regulated by IGFs, which may contribute to a deeper understanding of ovarian development and reproduction in teleosts, with implications for promoting genetic breeding for aquaculture development [23,26,55].

### 4.1. Differentially Expressed Genes and Their Regulatory Pathways

In this study, multiple differentially expressed genes encoding key enzymes involved in progesterone-mediated oocyte maturation and oocyte meiosis pathways, including *cyp17a1*, *cyp19a1*, *star*, *hsd17b3*, and *hsd17b7*, were identified. These genes are predominantly expressed in response to IGF1 stimulation during stage III of ovarian development, a phase marked by significant ovarian growth and maturation, as in most teleost species [37,56]. For instance, in *Cynoglossus semilaevis*, steroid hormones *cyp17a1*, *cyp19a1*, and *hsd17b1* are highly expressed during stage V of ovarian development when oocytes reach full maturation [24,57]. However, their expression significantly decreased as oocytes transitioned from stages V to VI, suggesting that these genes play a pivotal role in stage III ovarian development and oocyte maturation [58,59]. In female rainbow trout, treatment with IGF2 upregulates *star* mRNAs, which encode ovarian steroidogenic enzymes [13,32]. These findings suggest that IGFs play a regulatory role in ovarian development and maturation by influencing steroid hormones production.

In both mammals and teleosts, oocyte meiotic division is essential for oocyte maturation. In fish, *Foxn5* and *foxl2* have been reported to be associated with the oocyte meiosis pathway along with the FoxO and TGF- β signaling pathways at different stages of ovarian development [52]. Studies have indicated that *foxn5* and *foxl2* play a critical role in regulating ovarian differentiation and development in vertebrates [36,49,53]. Foxl2 is primarily found in the granulosa cells and early ovarian stroma of vertebrates, and its knockdown leads to disorganized ovarian follicular development and, in some cases, partial ovary-to-testis sex reversal [50,60,61]. In *Crassostrea gigas*, *foxl2* expression is significantly higher in mature females than in mature males, suggesting its involvement in vitellogenesis or female sex determination [62,63]. Previous studies in species such as spotted scats [61], rainbow trout [50], zebrafish [60], and Japanese flounder [50] have consistently demonstrated that *foxl2* exhibits significant sex dimorphic expression patterns and plays an important role in gonadal differentiation and development in fish. In common carp, RNA interference targeting *foxl2* leads to upregulation of *cyp19b*, emphasizing its regulatory role in ovarian development [64]. In the present study, in vitro treatment of *T. ovatus* ovaries with IGF1 and IGF2 upregulated *foxl2* and *foxn5*, respectively.

Extracellular matrix (ECM) receptor interactions have been shown to play a significant role in cell cycle processes during ovarian remodeling, including follicular formation and ovulation [24,65]. The matrix metalloproteinase (MMP) family serves as the primary enzyme responsible for ECM remodeling [24]. In the present study, we identified several DEGs related to the reproductive cycle. Specifically, *cdk15* and *cdk14* were differentially expressed after IGF1 stimulation, whereas *cdk5*, *cdk6*, *cd209*, and *ccnk* were differentially expressed after IGF2 stimulation during stage III ovarian development. These genes are associated with the cell cycle pathway, which is essential for ovarian maturation. The cyclin-dependent kinase (*cdk*) model has become a cornerstone for understanding and studying cell cycle regulation [24,31,32,66].

In tongue sole, many DEGs related to cell adhesion have been identified at various stages of ovarian development, including krt18 [24,67]. Consistent with these findings, our study revealed a significant expression of cell adhesion-related genes, such as krt8 and krt18, in the ovary in response to IGF1 stimulation. This highlights the critical role of endocrine hormones in T. ovatus reproduction. Furthermore, the ECM-receptor interaction and focal adhesion pathways were enriched in both IGF1 and IGF2 treatment groups. These findings suggest that endocrine hormones are essential for reproductive processes.

Studies have explored the regulation of the Wnt signaling pathway in follicle development and corpus luteum (CL) formation [68,69]. Wnt signaling has been shown to promote follicular growth and mature oocyte production by influencing cell proliferation, differentiation, stem cell renewal, mortality, and apoptosis, as well as maintaining tissue homeostasis during embryogenesis [69,70,71]. *β*-catenin activation is strongly associated with the transition of primordial follicles to growing follicles, highlighting its role in follicular activation [69]. In rainbow trout, *wnt11* and *wnt9b* are highly expressed in granulosa cells and oocytes during the later stages of ovarian development, highlighting their roles in folliculogenesis and oogenesis [46]. Similarly, in catfish, *wnt4* and *wnt5* are implicated in ovarian follicle development and play a key role in gonadal differentiation and maturation [46]. In the present study, *wnt6* and *wnt8* were significantly enriched in the ovary, which is consistent with findings from previous research. These results suggest that Wnt signaling pathways play an important role in regulating ovarian growth, development, and maturation, particularly in response to IGF treatment. This highlights the intricate interplay between the Wnt signaling pathway and IGF in supporting ovarian development and reproductive success.

The PI3K-Akt signaling pathway plays a pivotal role in regulating oocyte growth by controlling cell survival, metabolism, and nutrient uptake, thereby facilitating oocyte differentiation [19,72,73]. These integrated circuits ensure that ovarian maturation occurs at the right time and progresses correctly, which is critical for successful reproduction. The role of Akt in phosphorylating substrates involved in cell cycle progression helps ensure the proper maturation of oocytes [73]. Akt regulates metabolic processes by regulating mammalian target of rapamycin complex 1 (mTORC1), which is essential for protein synthesis and energy metabolism in oocytes [74,75]. In zebrafish, IGF1 enhances mitochondrial bioenergetics, boosting the ATP production required for oocyte maturation [12]. Dysregulation of the PI3K-Akt signaling pathway can impair reproductive potential, emphasizing its potential in maintaining oocyte health. In the present study, IGF treatment enriched the PI3K-AKT signaling pathway with genes, such as Tubulin Tyrosin Ligase-like 3 (*ttll3)*, Kelch-like protein 11 *(klhl11)*, KN Motif and Ankyrin Repeat Domain-Containing Protein 1 *(kank1)*, and Insulin-like Growth Factor Binding Protein 6 *(igfbp6)*, that are involved in regulating reproductive success and ensuring oocyte maturation.

### 4.2. Co-Expression and Tissue Distribution Analysis

Similar to the DE mRNAs, we identified several lncRNAs and target genes enriched in the same biological processes and pathways, including the cell cycle, oocyte meiosis, focal adhesion, and signaling pathways such as the MAPK, PI3K-Akt, GnRH, Wnt, FoxO, and Estrogen signaling pathways, as discussed previously in this study. These pathways regulate a wide range of physiological processes. Among the identified lncRNAs, four lncRNAs (MSTRG.66521.1, MSTRG.49969.1, MSTRG.59923.1, and MSTRG.13767.1) in response to IGF1 stimulation and two lncRNAs (MSTRG.20896.2, and MSTRG.58123.2) in response to IGF2 stimulation, are directly co-regulated with several DE mRNAs associated with ovarian development and maturation, including *adam23*, *cyp19a1*, *cyp17a*, *slc26a6*, *htr2b*, *h2ax*, *nanos3*, *krt18*, *pgr*, and *inhbb*, as shown in Figure 8 and Figure 9.

Specifically, *inhbb* and *pgr* were targeted by MSTRG.58123.3, while *nanos3* and *h2ax* were regulated by MSTRG.20896.2 in response to IGF2 treatment. Interestingly, in zebrafish, the expression of *inhbb* remained relatively unchanged during follicle development but increased sharply in the ovary before spawning [76,77]. Moreover, we observed that these *trans-target* genes were enriched in the cell cycle and TGF-β signaling pathways. Members of the TGF-β family, including inhibin and activin, are essential for follicular development and steroidogenesis [77]. Disruption of PGR in zebrafish has been shown to result in female infertility due to unsuccessful ovulation, raising intriguing questions about whether the absence of PGR might also affect early follicle growth [24]. Furthermore, in common carp, the TGF-β signaling pathway plays a pivotal role in regulating lipids and vitellogenesis during ovarian growth [24,78]. These findings underscore the importance of these molecular mechanisms in regulating ovarian development and provide valuable insights for further exploration of the reproductive processes in fish.

Interestingly, treatment with IGF1 upregulated several lncRNAs, including MSTRG.66521.1, MSTRG.49969.1, MSTRG.59923.1, and MSTRG.13767.1, which target key genes, such as *cyp19a1*, *cypq7a1*, *dmc1*, *eno1*, *htr2b*, and *csmd3*. These genes are enriched in ECM receptor interaction and the Estrogen, MAPK, and PI3K-Akt signaling pathways [24]. Dysregulation of these pathways can lead to conditions such as polycystic ovary syndrome, which negatively affects fish fertility and oocyte maturation. Gonadotropin-releasing hormone (GnRH) plays a vital role in stimulating the release of gonadotropins, which are critical for initiating oocyte maturation [79]. In tilapia, GnRH signaling is linked to the timing of ovulation and the reproductive cycle. Similarly, in *Cynoglossus semilaevis*, specific lncRNAs (XR_522498.1 and XR_522499.1) and their target genes were enriched in the TGF-β and Wnt signaling pathways [24]. These findings are consistent with our results and further support the importance of these pathways in ovarian development and oocyte maturation.

Real-time quantitative PCR analysis of tissue distribution revealed that all candidate lncRNAs were highly expressed in the brain, pituitary gland, liver, and gonads of golden pompano. These findings, similar to those of previous studies [23,32,63], suggest that selected lncRNAs may play critical roles in regulating the growth and reproductive axis by regulating their target genes. Further studies involving functional validation, such as knockdown or overexpression of these lncRNAs, are necessary to elucidate their roles and effects on ovarian development and maturation.

## 5. Conclusions

In this study, we utilized RNA-seq technology to investigate lncRNA–mRNA regulatory mechanisms associated with the ovarian development of *Trachinotus ovatus* under IGF1 and IGF2 stimulation during stage III of ovarian development. According to the lncRNA–mRNA network, we found six lncRNAs, such as MSTRG.66521.1, MSTRG.49969.1, MSTRG.59923.1, MSTRG.13767.1, MSTRG.20896.2, and MSTRG.58123.2, that associated with the most of DEGs involved in ovarian development and oocyte maturation including *cyp17a1*, *cyp19a1*, *star*, *hsd17b3*, *hsd17b7*, *dmc1*, *eno1*, *htr2b*, *csmd3*, *slc26a6*, *htr2b*, *h2ax*, *nanos3*, *krt18*, *pgr*, and *inhbb*. These genes were associated with key pathways, such as oocyte meiosis, ECM receptor interaction, Estrogen, Wnt, FoxO, MAPK, TGF-β, GnRH signaling pathways, and focal adhesion. Tissue distribution analysis revealed that these candidate lncRNAs were dominantly expressed in the gonads, suggesting they may play critical roles in *T. ovatus* reproduction by regulating gene expression. The findings of this study provide insights into potential molecular mechanisms by which lncRNAs regulate mRNA expression in response to IGF stimulation that may regulate ovarian development and oocyte maturation. This study establishes a foundational framework for advancing targeted breeding strategies in golden pompano aquaculture and offers a model for understanding reproductive regulation in teleosts.

## Figures and Tables

**Table 1:** IGF1 and IGF2 primer sequences of the lncRNAs and mRNAs for the RT-qPCR analysis.

ID Description	Gene Name	Primers (5′–3′)
SCSFRI_TO_T_00091676	*cyp19a1*	F: TGGCTCATGTGGATGTCCTCAGT
		R: TGCTGTCCTGTGCTGCTGGTTA
SCSFRI_TO_T_00132967	*cyp17a1*	F: ACGCTCTGCTTCAACTCCTCCT
		R: AAGATGTCCACCAGGCTGTCCTT
SCSFRI_TO_T_00065781	*adam23*	F: GTGGTGCTCGTTGCTGTGGA
		R: GGCGTGGTGCTTGATGCTCT
SCSFRI_TO_T_00083864	*hsd17b7*	F: AGCCAGCTTCGCCTGTGTCT
		R: ACCTCCTGAGCAGCAGTGAACA
SCSFRI_TO_T_00068502	*hsd17b3*	F: TGGTGACTGGTGCTTCAGAAGG
		R: TGGCTACCTGGTCCAATGTTACTT
SCSFRI_TO_T_00052923	*sox3*	F: GAGCCAACGCCGTGAACAACT
		R: GTAGGTCTGAGCCGAGGACATCA
SCSFRI_TO_T_00073261	*foxn5*	F: ACGCTGGGAGCCTCAAAGTC
		R: CAGGTTGTGTCGGATGGTGTTC
SCSFRI_TO_T_00072390	*inhbb*	F: GCAACGAGGTTCTAGCGGAGAC
		R: ACACGGAAGTAGAGCCACAGGTT
SCSFRI_TO_T_00126075	*krt18*	F: ACCGCATCAGCATCTCCTCCA
		R: CTCCAGGTTCCTCACTGTCTCCA
SCSFRI_TO_T_00025866	*h2ax*	F: GTCGGTCGTGTTCACAGGCT
		R: CCAGGATCTCAGCGGTCAGGTA
SCSFRI_TO_T_00061632	*nanos3*	F: ACCGCAAGAAGACGCCCAAG
		R: CACAACACATCTCCTGCCTGGT
SCSFRI_TO_T_00003998	*kank1*	F: TGGCTCCAACAAGGCAACGAA
		R: GGCTGGACACAGAGAACCACTC
SCSFRI_TO_T_00026350	*slc6a18*	F: CCCTGTGCTTTCCATCCCTCTG
		R: CGGTTCCTCTGTTCCTGTGCTT
SCSFRI_TO_T_00012814	*pgr*	F: GTTCAGAGGACGAATGTGGCTACG
		R: TGCTGCTTCCTGTCGGTGTCA
SCSFRI_TO_T_00113132	*cdk15*	F: GCTGGTGGCTCTGAAGGTGATC
		R: AGTCTCTGGCGTGGATGATGTCA
MSTRG.57329	*star*	F: GAGGATGGATACAGCGAGGAGGAG
		R: CCAATGTCAGGCAACACCTTACTCA
SCSFRI_TO_T_00138572	*axin2*	F: CGCAGAGAGTCGCCAAAGCAAT
		R: TCCAGGTAGATGTCAGAGGTCAGAA
SCSFRI_TO_T_00073261	*β-actin*	F: GAGAGGTTCCGTTGCCCAGAG
		R: CAGACAGCACAGTGTTGGCGT
MSTRG.26614.1		F: AACATCACGGCTCTTGGCTCTC
		R: GCTTGTGCTGGAGGCTGAAGT
MSTRG.98390.1		F: CGCTGGCATTGGTCACATTCAC
		R: GAGAACGGTGTCCACTCCATCG
MSTRG.77400.2		F: GACATGCCTGCTCTGTGATTGA
		R: CCACTCTGTCAGTACCGAGGAA
MSTRG.84457.1		F: ATCGCCTTCCGTCCCTTCCA
		R: CCTCGCCTGCTGTTTGGTCAT
MSTRG.20896.2		F: TGTCTGCCTGCTCCACATCCT
		R: TGTCCTCCCAGCCCTGTCATT
MSTRG.58123.2		F: AGATAAGGAGTGCAGCCTGTGT
		R: CGCAAGGAGAACGGACAGAGA

**Table 2:** Summary of the reads after quality control.

Sample	Clean Read Bases	Q20 (%)	Q30 (%)	Total Clean Reads	Mapped Reads	Mapping Ratio (%)
**C1**	15,630,440,161	98.17	94.96	1.11 × 10^8^	1,332,878	91.75
**C2**	13,264,457,783	98.37	95.34	94,414,990	1,897,056	90.71
**C3**	12,982,764,898	98.16	94.91	91,848,966	1,367,594	91.24
**IGF1T1**	13,110,122,504	98.31	95.26	93,054,020	1,202,186	92.21
**IGF1T2**	13,516,459,173	98.25	95.13	98,391,424	3,977,422	90.13
**IGF1T3**	12,471,670,121	98.38	95.44	91,910,782	3,211,596	91.53
**IGF2T1**	13,269,497,776	98.33	95.28	97,543,940	1,117,334	92.94
**IGF2T2**	14,829,071,350	98.29	95.14	1.09 × 10^8^	971,076	93.06
**IGF2T3**	14,431,477,858	98.42	95.47	1.05 × 10^8^	1,243,074	93.27

Note: C refers to the control group, and T refers to the treated group.

## Data Availability

The data supporting this study’s findings are available upon reasonable request. The raw reads in this article have been deposited into the Sequence Read Archive (SRA) of the NCBI database under BioProject accession number: PRJNA1224432.

## Supplementary Materials

The following supporting information can be downloaded at: https://www.mdpi.com/article/10.3390/ani15081134/s1, Table S1. Primer sequences for amplifying IGF1 and IGF2 genes used in recombinant protein expression. Table S2. Differentially expressed lncRNAs and mRNAs information in the control versus treated groups. Table S3. GO enrichment analysis of DE lncRNAs and DE mRNAs in the control versus treated groups. TableS4. KEGG pathway enrichment analysis of DE lncRNAs and DE mRNAs in the control versus treated groups.
