# Supplementary material for: Integrated lncRNA and mRNA Transcriptome Analyses of IGF1 and IGF2 Stimulated Ovaries Reveal Genes and Pathways Potentially Associated with Ovarian Development and Oocyte Maturation in Golden Pompano (Trachinotus ovatus)
Source: Animals (Basel). 2025 Apr 15;15(8):1134. doi: 10.3390/ani15081134 (PMC12024298; doi:10.3390/ani15081134)
Supplement: Supplementary file 1 [file animals-15-01134-s001.zip › Table S1.pdf]

Table S1: Primer sequences for amplifying IGF1 and IGF2 genes used in recombinant protein expression

Gene	Purpose	Primer	5′ to 3′ Sequence
**IGF1**	Recombinant protein	IGF1F	ggtggtggatccgaattccggactATGGCAGGTCCGGAAACCCTGTGCGGCGCAGAACTGGTTGATACCCTGCAGT
		IGF1R	ggtggtggtgctcgagtgcggccttaTTACATACGATAATTACGACCGCCGGTATTGCCACGACTGCTATTTTTC
**IGF2**	Recombinant protein	IGF2F	tgggatccggtaccaagcttGAGACGCTGTGTGGGGGAGA
		IGF2R	tggtggtggtggtgctcgagTCATTTGTGGTTGACATAGTTG
